# Comparative insights into posthumous organ donation attitudes in chronically ill and healthy Egyptians

**DOI:** 10.1038/s41598-025-23588-6

**Published:** 2025-11-07

**Authors:** Ammal M. Metwally, Safaa I. Abd El Hady, Hend I. Salama, Ghada A. Elshaarawy, Raefa R. Alam, Wafaa M. Elboraey, Zeinab M. El-Bouraey, Hanan M. Badran, Hanan M. Mohamed, Mohamed Abdelrahman, Amira S. ElRifay, Shereen M. El Khateeb, Abdelrahman K. Hassanein

**Affiliations:** 1 Community Medicine Research Department, Medical Research and Clinical Studies Institute, National Research Centre, Dokki, Cairo, Egypt; 2 Department of Psychiatry, Mansoura General Hospital, Ministry of Health and Population, Al Dakahlyia, Egypt; 3 National Blood Transfusion Services Mansoura Region, Ministry of Health and Population, Al Dakahlyia, Egypt; 4 Faculty of Nursing, Mansoura National University, Gamasa, Egypt; 5 Technical Institute of Nursing, Sherbin, Al Dakahlyia, Egypt; 6 Child Health Department, Medical Research and Clinical Studies Institute, National Research Centre, Dokki, Cairo, Egypt; 7 Department of Psychology, American University in Cairo, Cairo, Egypt; 8 Community Medicine Research Department, Medical Research and Clinical Studies Institute, National Research Centre, Dokki, Giza, Egypt

**Keywords:** Organ donation, Egypt, Posthumous donation, Public health, Cultural beliefs, Legal considerations, Health care, Medical research

## Abstract

Organ transplantation is a life-saving intervention, yet a persistent global organ shortage threatens patient survival. In Egypt, cultural, religious, and legal factors significantly influence donation willingness, contributing to persistently low consent rates. This study aimed to: (1) compare attitudes toward posthumous organ donation between patients with chronic illness and healthy individuals; (2) assess their preferences for eleven distinct consent models; and (3) evaluate awareness of Egyptian organ donation laws and how this and key socio-demographic predictors influence willingness to donate. This cross-sectional study included 6,000 participants (3,000 patients and 3,000 healthy individuals) from two Egyptian governorates. Participants were selected through stratified random sampling. Structured interviews and self-administered questionnaires collected data on socio-demographics, donation knowledge, and consent preferences. Participants ranked eleven consent models and assessed their awareness of key Egyptian organ donation laws. Patients exhibited a significantly higher willingness to donate posthumously (91%) compared to healthy individuals (60%) (*p* < 0.01). Written consent was preferred over verbal consent in both groups (75.2% vs. 70.1%, *p* < 0.001), emphasizing the importance of formal documentation. Incentivized donation was more favored by patients (59.6%) than healthy participants (54.7%) (*p* = 0.001), indicating incentives could enhance participation. Among patients, chronic illness was the primary motivator for donation, rendering socio-demographic variables less relevant. However, logistic regression identified key predictors among healthy participants. Participants aged 45–65 years were significantly less willing to donate than those over 65 years (AOR = 0.41, *p* = 0.01). Those in education (AOR = 2.07, *p* = 0.006) and manual workers (AOR = 1.94, *p* = 0.004) were more likely to donate than the unemployed. Higher socioeconomic status (C1/C2 and A/B) was associated with lower donation willingness than the lowest SES (D) (AOR = 0.34, 0.31, *p* < 0.001). Greater legal awareness was significantly associated with higher willingness to donate (*p* < 0.001). Chronic illness enhances donation willingness independent of socio-demographics. Health authorities should implement adaptive consent frameworks to enhance organ donation rates in Egypt, including simplified, surrogate-inclusive models for patients. Structured legal education programs for the general public to address knowledge gaps are recommended.

## Introduction

Organ transplantation is a vital, life-saving intervention for patients suffering from end-stage organ failure, significantly enhancing both survival rates and quality of life. Despite advances in medical technology and increased global efforts to promote organ donation, the shortage of transplantable organs remains a major challenge. This shortage leads to prolonged waiting lists and high mortality rates among patients in need of transplantation^1^.

In Egypt, kidney transplantation is the most commonly performed procedure, with organ transplantation practices spanning over four decades^2,3^. The growing burden of chronic heart failure has similarly escalated the demand for heart transplants, placing additional strain on the healthcare system and highlighting the need for regulatory clarity and more efficient donation mechanisms^4^. However, religious, cultural, and legal barriers continue to restrict the number of transplants performed, limiting access to life-saving treatment^5^.

Ethical and cultural issues in Egypt; particularly those involving brain death criteria and consent are deeply influenced by religious beliefs and family roles in decision-making. In many cases, family members may override a deceased individual’s prior decision to donate due to cultural and religious concerns^6,7^. Moreover, misconceptions regarding organ donation; such as fears about bodily integrity and distrust in the medical system contribute to public reluctance toward posthumous donation^8^. Religious beliefs, especially within Islamic contexts, significantly shape public attitudes. While many Islamic scholars support organ donation as an act of charity (sadaqah), divergent interpretations; particularly regarding brain death and consent continue to generate hesitation^9,10^. These religious ambiguities can contribute to family refusal and a lack of formal donor registration, despite general awareness of donation.

Globally, organ transplantation frameworks vary, with each country adopting different policies and regulations. In Egypt, stringent laws govern donor eligibility, requiring confirmation of death by a medical committee and imposing severe penalties for illegal organ trading^2,11^.

Although Egypt has a well-established history of kidney and liver transplantation^12^ and remains a major contributor to regional transplant activity, the demand for organs far exceeds supply, contributing to a rise in illegal organ trade; a phenomenon that poses serious ethical and health risks^5,11^. Data from the International Registry in Organ Donation and Transplantation (IRODaT) show that countries such as Turkey and Iran report significantly higher transplant rates per capita, particularly in kidney transplantation. Iran, in particular, has consistently ranked among the global leaders in living kidney donation and has implemented a regulated living-unrelated donor system with national legislative oversight^13,14^. Saudi Arabia has also demonstrated notable progress, with the King Faisal Specialist Hospital and Research Centre performing over 9,000 successful organ transplants since 1981. Notably, the hospital conducted the world’s first fully robotic liver transplant from a living donor in 2023 and the first fully robotic heart transplant on a 16-year-old patient with end-stage heart failure in 2024^15,16^.

These disparities highlight the pressing need for Egypt to reform its organ transplantation system. By studying the policies and initiatives adopted by neighboring countries, Egypt can identify effective strategies to address organ shortages and improve transplantation accessibility. Enhancing legal frameworks, promoting ethical procurement, and investing in public education could help increase donation rates and build public trust.

The Theory of Planned Behavior (TPB) and the Health Belief Model (HBM) are pivotal in understanding organ donation behaviors^17^. TPB posits that an individual’s intention to donate is influenced by their attitude toward donation, perceived social norms, and perceived behavioral control^18^. Research indicates that these factors significantly predict donation intentions and behaviors. Conversely, HBM suggests that individuals’ decisions to engage in health behaviors, such as organ donation, are shaped by their perceptions of susceptibility to health issues, the severity of these issues, the benefits of taking action, and potential barriers^19^. This model has been effectively applied to understand organ donation intentions across diverse populations. Combining TPB and HBM allows for a more holistic understanding of the psychological and social factors influencing donation behavior, enabling the design of more effective, evidence-based interventions^20^. Previous studies highlight the necessity of public awareness campaigns and educational initiatives to dispel myths and promote informed consent regarding organ donation^2,21,22^. However, limited research has explored attitudes toward organ donation across different demographic groups in Egypt, particularly among both healthy individuals and patients with chronic illness.

To address this gap, the current study aimed to: (1) compare attitudes toward posthumous organ donation between patients with chronic illness and healthy individuals; (2) assess their preferences for eleven distinct consent models; and (3) evaluate awareness of Egyptian organ donation laws and how this and key socio-demographic predictors influence willingness to donate. These findings aim to inform targeted public policies and ethical frameworks to enhance organ donation rates in Egypt.

## Methods

### Study Design, Setting, and participants

This cross-sectional study assessed the attitudes and willingness of Egyptians toward posthumous organ donation, comparing patients with chronic illness to healthy individuals. It is part of a broader project on social acceptance and consent preferences regarding posthumous organ donation in Egypt. Previous results from this project, focused on general consent preferences, have been published^2^.

The study was conducted in health facilities across two Egyptian governorates, Dakhlyia and Giza, selected for their diverse urban and rural populations, differing socioeconomic backgrounds, and accessibility to major medical centres.

Participants included 6,000 individuals: 3,000 patients with chronic illness diagnosed with hepatic or renal conditions; chronic illnesses commonly associated with end-stage organ failure and future need for transplantation and 3,000 healthy individuals. Healthy controls were chosen from close relatives due to their accessibility in the health facility setting.

### Behavioral theories guiding the study: theoretical integration between TPB and HBM

To operationalize the theoretical foundation of this study, we integrated TPB and HBM into our conceptual framework. TPB was used to explore how attitudes, social norms, and perceived control influenced willingness to donate among healthy individuals. Meanwhile, HBM helped interpret the motivations of patients with chronic illness, emphasizing their perceived health vulnerability and benefit recognition. This integrated framework allowed us to investigate how internal health experiences and external cognitive cues; such as legal awareness and cultural norms differentially shape donation intentions in both groups.

### Inclusion and exclusion criteria

Participants were selected based on inclusion and exclusion criteria to ensure the validity and reliability of the study findings. Patients with chronic illness were eligible for inclusion if they were adults (≥ 18 years old) diagnosed with hepatic (e.g., liver cirrhosis, hepatitis-induced liver disease) or renal disease (e.g., chronic kidney disease, end-stage renal failure) and were actively receiving treatment at one of the participating health facilities in either Dakhlyia or Giza. Patients with chronic illness were excluded if they were experiencing acute (rather than chronic) organ failure or were listed for immediate organ transplantation, as these circumstances could influence their views on donation in atypical ways. Additionally, patients were excluded if they exhibited severe cognitive impairment or mental health conditions that could compromise their capacity to give informed consent or reliably complete the survey.

Healthy controls were included if they were adults (≥ 18 years old) with no history of chronic illness and were first-degree relatives of patients with chronic illness. To validate their health status and ensure they were free from chronic diseases, each participant underwent a structured health screening process. This screening included a review of medical history, in which participants were asked about previous or current diagnoses of chronic conditions such as hepatic, renal, or cardiovascular diseases. Additionally, a basic clinical assessment was conducted, incorporating self-reported health status, recent hospital visits, and medication history. To further confirm eligibility, a qualified physician at the recruitment site reviewed the provided health information and, where necessary, performed brief clinical evaluations to rule out any underlying conditions. Healthy participants were excluded if the screening process identified undiagnosed chronic illnesses, if they were not first-degree relatives of patients, or if they had previously participated in similar organ donation studies, as prior exposure might have shaped their attitudes toward organ donation and introduced bias.

### Sample size

To detect a 2% difference in the acceptance of posthumous organ donation between patients with chronic illness and healthy individuals, we calculated the required sample size using a two-sided Z-test with pooled variance. The test was set at a significance level (α) of 0.01 and a power of 90%, ensuring sufficient sensitivity to detect even modest differences between the groups.

For the patient group, we expected a donation acceptance rate of 5% under the alternative hypothesis, compared to 3% under the null hypothesis. For the healthy group, the acceptance rate was assumed to be 3% under both hypotheses. Based on this, a minimum of 2,855 participants per group was required to achieve the desired power. To account for potential non-responses or dropouts, we aimed to recruit 3,000 participants per group.

**The equation is as follows** ^23–25^ **:**

**Two Independent Proportions (Null Case) Power Analysis** 

**Numeric Results of Tests Based on the Difference: P1 - P2**

**H0: P1-P2=0. H1: P1-P2=D1<>0. Test Statistic: Z test with pooled variance**

**Sample Sample Prop|H1 Prop**

**Size Size Grp 1 or Grp 2 or Diff Diff**

**Grp 1 Grp 2 Trtmnt Control if H0 if H1 Target**

**Power N1 N2 P1 P2 D0 D1 Alpha Beta**

 0.90 2855 2855 0.05 0.03 0.00 0.02 0.01 0.09

“Power” is the probability of rejecting a false null hypothesis (desired = 0.90). “N1 and N2” are the sizes of the samples drawn for the two groups. P1 and P2 are the expected proportions under H₁ for patients and controls, respectively. “Target Alpha” is the probability of rejecting a true null hypothesis that was desired. “Actual Alpha” is the value of alpha that is actually achieved. D1 is the expected difference (0.02), and α is the significance level. “Beta” is the probability of accepting a false null hypothesis.

## Sampling technique

Participants were recruited using a stratified random sampling technique to ensure that the sample accurately reflected the population of interest, particularly when comparing patients with chronic illness and healthy individuals.

Stratification was based on four key socio-demographic variables known to influence attitudes toward organ donation: age (categorized as under 25, 25–44, 45–64, and 65 years or older)^26^, Sex: (Male, Female), employment status (employed, unemployed, student, retired)^27^, and education level (no formal education, primary/preparatory, secondary, and higher education).

Population distributions for these strata were derived from recent census data and health facility records in Dakhlyia and Giza. Sampling proportions were set to match the demographic structure of each governorate. Within each stratum, participants were randomly selected using a computer-generated list, with equal representation from both governorates to ensure geographic balance.

Given the distinct urbanization and healthcare access levels in Dakhlyia and Giza, stratification was conducted independently in each governorate. Proportional adjustments ensured that demographic quotas aligned with local population characteristics. To minimize selection bias, quotas were double-checked during recruitment to prevent over- or under-representation of any subgroup. Participants were recruited sequentially from hospitals and healthcare facilities, with efforts made to balance participation between urban and rural areas.

## Tools and data collection

The data collection process involved both structured face-to-face interviews and self-administered questionnaires, which were completed by all participants included in the final study sample (*N* = 6,000). These tools were developed to capture basic socio-demographic characteristics along with nuanced personal preferences and attitudes toward posthumous organ donation.

The questionnaire was informed by an extensive review of relevant literature and consultations with experts in public health and organ transplantation. It was pre-tested on a pilot sample of 30 individuals to ensure clarity, cultural appropriateness, and internal consistency.

All participants received a detailed explanation of the study objectives and instructions before completing the questionnaire. Before completing the questionnaire, all participants received a detailed explanation from the research team about the study’s purpose, the structure and content of the questionnaire, and the definitions of key terms related to organ donation. This orientation ensured that even those unfamiliar with the subject could provide informed responses. Literate participants completed the questionnaire independently following this briefing. For illiterate participants, trained research staff conducted structured verbal interviews, reading each item aloud and recording the responses to ensure accuracy and completeness.

The final sample size of 6,000 participants represents 95.2% of those initially approached, after excluding 4.8% who were either ineligible based on screening criteria or did not complete the required forms. This high response rate enhances the representativeness and reliability of the findings.

The data collected included:

- **Demographic Data**^27^: This encompassed a wide range of socio-demographic variables such as age, sex, education level **(**from illiterate to college or higher), occupation (including professional, technical, managerial, skilled, and unskilled manual workers, as well as unemployed or in education), and.
- Sociodemographic factors, including sex, age, education level, occupation, and socioeconomic status (SES), were analyzed to assess their influence on willingness to donate. The Egyptian SES classification system categorizes families into four groups (A, B, C, D) based on seven domains^27^: education and cultural exposure, occupation, family structure, asset ownership, income stability, home sanitation, and healthcare access. Class A represents the highest SES group with superior education, prestigious jobs, and financial stability, while Class D reflects the lowest SES group, characterized by lower education, unstable employment, and limited resources. This classification provides a nuanced understanding of social disparities, supporting targeted policies.

The Socioeconomic Status (SES) Categorization allows for the assessment of health disparities and the impact of socioeconomic factors on health. This stratification is in line with standard practices in assessing social determinants of health. Categories included8: Class A (Highest SES - Upper Class/Elite), Class B (Upper-Middle SES), Class C (Lower-Middle SES) and Class D (Lowest SES - Economically Disadvantaged). Class C can be further classified to Class C1 (Lower-Middle SES - Skilled Workers e.g. technicians, teachers, government employees) and Class C2 (Lower-Middle SES - Semi-Skilled Workers e.g. factory workers, drivers, low-ranking administrative employees).

In addition to socio-demographics, data collected included participants’ personal familiarity with organ donation, preferences for donation consent models, and awareness of existing Egyptian organ donation laws.

## Consent preferences and their application in the study

In the context of posthumous organ donation, different consent models reflect varying levels of individual autonomy, state involvement, and incentivization. The current study examined eleven consenting options, adapted from Hammami et al.^28^, allowing participants to rank their preferences. The following definitions and applications were presented to participants:

## • no organ donation

### Definition

Individuals explicitly refuse to donate their organs after death (posthumous donation), either by personal choice or due to cultural/religious reasons.

### Application in the study

Participants could rank this option to indicate complete opposition to posthumous organ donation.

- **Presumed Consent** (Opt-Out System).

### Definition

Individuals are automatically considered organ donors upon death unless they actively register to opt out during their lifetime.

### Application in the study

Participants were asked whether they support or oppose presumed consent **as a** default policy in Egypt.

### Informed consent

• **by Donor (Without Incentives)**.

### Definition

Individuals must actively register as organ donors without receiving any compensation or benefit.

### Application in the study

Participants indicated whether they prefer voluntary donation without incentives.

### Informed consent

• **by Donor (With Medical or Financial Incentives)**.

### Definition

Individuals voluntarily register as donors and receive benefits such as transplant list priority or financial compensation.

### Application in the study

Participants ranked whether they would be more willing to donate if such incentives were offered.

### Informed consent

• **by Donor or Surrogate (Without Incentives)**.

### Definition

Organ donation requires explicit consent either from the donor before death or from their next-of-kin after death, with no financial or medical incentives involved.

### Application in the study

Participants ranked whether families should be allowed to authorize donation when the deceased had not documented a preference.

### Informed consent

• **by Donor or Surrogate (With Medical or Financial Incentives)**.

### Definition

Either the donor or surviving relatives may consent to donation, with possible incentives for the family.

### Application in the study

Participants ranked whether financial or medical incentives would influence family decisions on organ donation.

## • mandatory choice (Without Incentives)

### Definition

The law requires individuals to state their organ donation preference during official procedures (e.g., driver’s license application), without receiving any incentives.

### Application in the study

Participants ranked whether mandatory declaration without incentives would be acceptable.

## • mandatory choice (With medical or financial Incentives)

### Definition

Individuals must state a donation preference, and those who consent receive benefits such as healthcare priority or tax deductions.

### Application in the study

Participants ranked whether linking mandatory choice with incentives would increase willingness to donate.

## Egyptian organ donation law: awareness of key articles

Participants were asked whether they were aware of each of the seven key articles governing organ donation under Egyptian law, using a structured yes/no response format followed by a brief description of each article to ensure comprehension as follows:

### Organ donation between children of mixed nationality

The law permits organ donations between children of an Egyptian mother and a foreign father, ensuring eligibility for transplants despite differences in nationality.

- **Verification of Death Prior to Organ Donation**: Organ or tissue transfer from a deceased individual is prohibited until death is confirmed by a triple medical committee consisting of specialists in neurosurgery, cardiothoracic surgery, and anaesthesia.
- **Organ Donation to Non-Relatives in Urgent Cases**: Organ donations to non-relatives are permissible if the recipient is in urgent need of a transplant and meets the necessary medical criteria.
- **Government Coverage of Transplant Expenses**: For those who cannot afford the costs, the Egyptian government covers the expenses of organ transplantation operations in licensed medical facilities, following regulations set by the Ministry of Health.
- **Living Donor Transplantations Restricted to Relatives**: Organ transplants between living individuals are allowed only for donations among relatives who are Egyptian nationals, ensuring donations remain within family lines unless in critical circumstances.
- **Penalties for Illegal Commercial Transplants**: Severe penalties, including long jail sentences and substantial fines, are imposed on individuals involved in commercial organ transplants that violate any part of the law.
- **Premeditated Murder Penalty for Illegal Organ Transfer**: Under Article 230, anyone who transfers an organ or tissue from a person without confirming death, as outlined in Article 14, is subject to the penalty for premeditated murder.

### Ensuring validity and reliability of the questionnaire

To guarantee the validity and reliability of the questionnaire assessing public awareness and acceptance of organ donation laws, the study followed a rigorous multi-step process. First, a panel of experts, including legal professionals, public health specialists, and sociologists, conducted an extensive review to ensure that each question was directly relevant to its corresponding legal provision and aligned with the study’s objectives. This expert validation helped refine the content, ensuring conceptual clarity and legal accuracy.

Next, pilot testing was conducted with a diverse group of 30 participants representative of the target population. This phase aimed to identify ambiguities, assess clarity, and enhance user-friendliness. Feedback from participants led to minor modifications, ensuring that the questionnaire was easily comprehensible and culturally appropriate.

To further assess internal consistency and reliability, the study applied Cronbach’s alpha with a larger sample of 60 participants, achieving high reliability values (≥ 0.8), indicating strong internal coherence among questionnaire items. This comprehensive validation process ensured that the final questionnaire was both methodologically sound and effective in capturing public perceptions of organ donation laws, providing a robust foundation for the study.

## Statistical analysis

All collected data were entered into IBM SPSS Statistics (version 28.0) for analysis. Descriptive statistics, such as frequencies and percentages, were used to summarize the data, providing a clear overview of participants’ responses. Bar charts were also used to visually represent key findings.

Data analysis was conducted based on participants’ sex, education level, employment status, and socioeconomic level, with a specific focus on comparing the chronically ill group with the healthy control group. The χ²-test was used to compare qualitative variables between groups, allowing for the identification of statistically significant differences in organ donation preferences.

Logistic regression analysis was employed to examine the independent effects of various socio-demographic factors on participants’ willingness to donate organs posthumously. This method is particularly well-suited for analyzing binary outcomes, such as whether an individual is willing or unwilling to donate, by estimating the probability of an event occurring while accounting for multiple predictors simultaneously. To ensure robust and unbiased estimates, the analysis adjusted for potential confounders, including: age, sex, education level, employment status, socioeconomic class, and awareness of organ donation laws.

The Adjusted Odds Ratio (AOR) was used as the primary measure of association, indicating how the likelihood of willingness to donate changes with each predictor while holding other factors constant. A confidence interval (CI) of 95% was reported for each AOR, with statistical significance determined at *p* ≤ 0.05. If the CI does not include 1.0, the association is considered statistically significant at *p* ≤ 0.05. This is because an AOR of 1.0 indicates no difference in likelihood between the comparison and reference groups. AOR > 1.0: Indicates that the group has a higher likelihood of willingness to donate compared to the reference group. AOR < 1.0: Indicates that the group has a lower likelihood of willingness to donate compared to the reference group. CI including 1.0: Suggests no statistically significant association, meaning that differences observed could be due to chance.

By applying logistic regression, the study was able to differentiate between significant and non-significant predictors, ensuring that observed relationships were not confounded by other variables.

### Handling of missing data

To compensate for potential participant losses, the study account for 5% extra cases for ensuring a robust sample. Then, a structured approach was implemented to identify, classify, and address missing data, ensuring data integrity and minimizing bias. Initial screening was conducted through double-entry verification into IBM SPSS Statistics (version 28.0) to identify inconsistencies and missing values. This was followed by a preliminary completeness check immediately after data entry to detect missing responses before beginning statistical analysis.

Statistical adjustments and sensitivity analysis were applied. Weighting adjustments were applied if missing data were significantly associated with demographic characteristics, ensuring that non-response bias did not distort findings. Sensitivity analysis compared results between complete-case analysis (excluding missing values). Logistic regression models included an indicator variable for missing data to assess whether exclusion biased the results.

## Results

The results section presents the findings on the socio-demographic characteristics of the participants, their awareness of organ donation laws, and their personal preferences for consenting to organ donation. The analysis revealed no statistically significant differences in the socio-demographic characteristics of the participants, as shown in Table 1, indicating that the groups were balanced and comparable. Both groups had similar age distributions, with nearly half of participants in the 25–45 years category (46.0% of patients vs. 47.0% of healthy participants, *p* = 0.20). The proportion of males and females was nearly equal between groups (45.0% male vs. 55.0% female in patients, 43.0% male vs. 57.0% female in healthy participants, *p* = 0.12). No significant differences were observed in employment categories. A substantial proportion of the employed participants engaged in manual work (75.5% of patients vs. 78.3.0% of healthy participants, *p* = 0.05). The majority had a secondary education, with similar distributions across different education levels (*p* = 0.20). Nearly equal proportions were found across all socioeconomic classes, with no statistical significance (*p* = 0.87).

Very few participants personally knew an organ donor (1.5% patients vs. 1.4% healthy, *p* = 0.746) or recipient (1.8% patients vs. 2.3% healthy, *p* = 0.147).

**Table 1:** Socio demographic data of the study participants.

Variables	Patients (*n* = 3000) *N* (%)	Healthy participants (*n* = 3000) *N* (%)	*P* value#
**Age** < 25 years 25-<45 years 45-<65 years > 65 years	750 (25.0) 1380 (46.0) 357 (11.9) 513 (17.1)	780 (26.0) 1410 (47.0) 357 (11.9) 453 (15.1)	0.20
**Sex** Male Female	1350 (45.0) 1650 (55.0)	1290 (43.0) 1710 (57.0)	0.12
**Employment** Employed Unemployed Student Retired	1590 (53.0) 357 (11.9) 540 (18.0) 513 (17.1)	1610 (53.7) 357 (11.9) 580 (19.3) 453 (15.1)	0.15
**Job (out of Employed)** Manual labour/construction Professional technical/managerial Public sector	1200 (75.5) 39 (2.5) 351 (22.0)	1260 (78.3) 48 (3.0) 302 (18.7)	0.05
**Education level** No formal education Primary/Preparatory Secondary University and Postgraduate	417 (13.9) 363 (12.1) 1350 (45.0) 870 (29.0)	450 (15.0) 400 (13.3) 1330 (44.3) 820 (27.4)	0.20
**Socioeconomic level** A class B class C class D class	124 (4.1) 996 (33.2) 1090 (36.3) 790 (26.4)	114 (3.8) 669 (33.2) 1080 (36.0) 810 (27.0)	0.87
**Know organ donor** Yes No	45 (1.5) 2955 (98.5)	42 (1.4) 2958 (98.6)	0.75
**Know organ recipient** Yes No	54 (1.8) 2946 (98.2)	70 (2.3) 2930 (97.7)	0.15

# test of significance: x2: chi-square test between %; All p-values > 0.05; no statistically significant differences were observed.

**Fig. 1:**
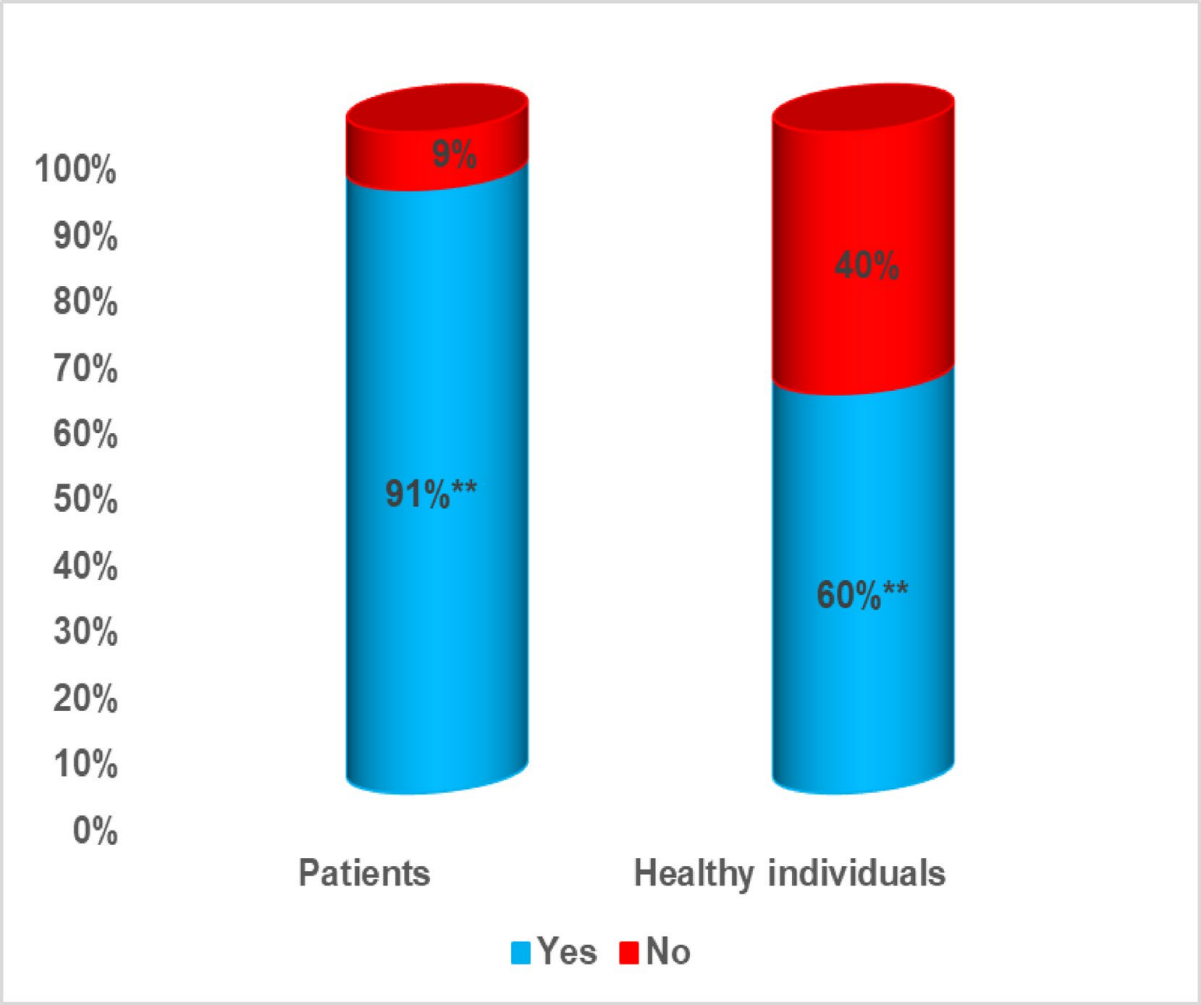
Acceptance of the concept of post-humous organ donation among patients with chronic illness and healthy controls. Test of significant was z test between proportions, ** Highly significant: *p* < 0.01.

Regarding the acceptance of posthumous organ donation (Fig. 1), a significant difference was observed, with a much higher acceptance rate among patients (91.0%) compared to healthy participants (60.0%).

**Fig. 2:**
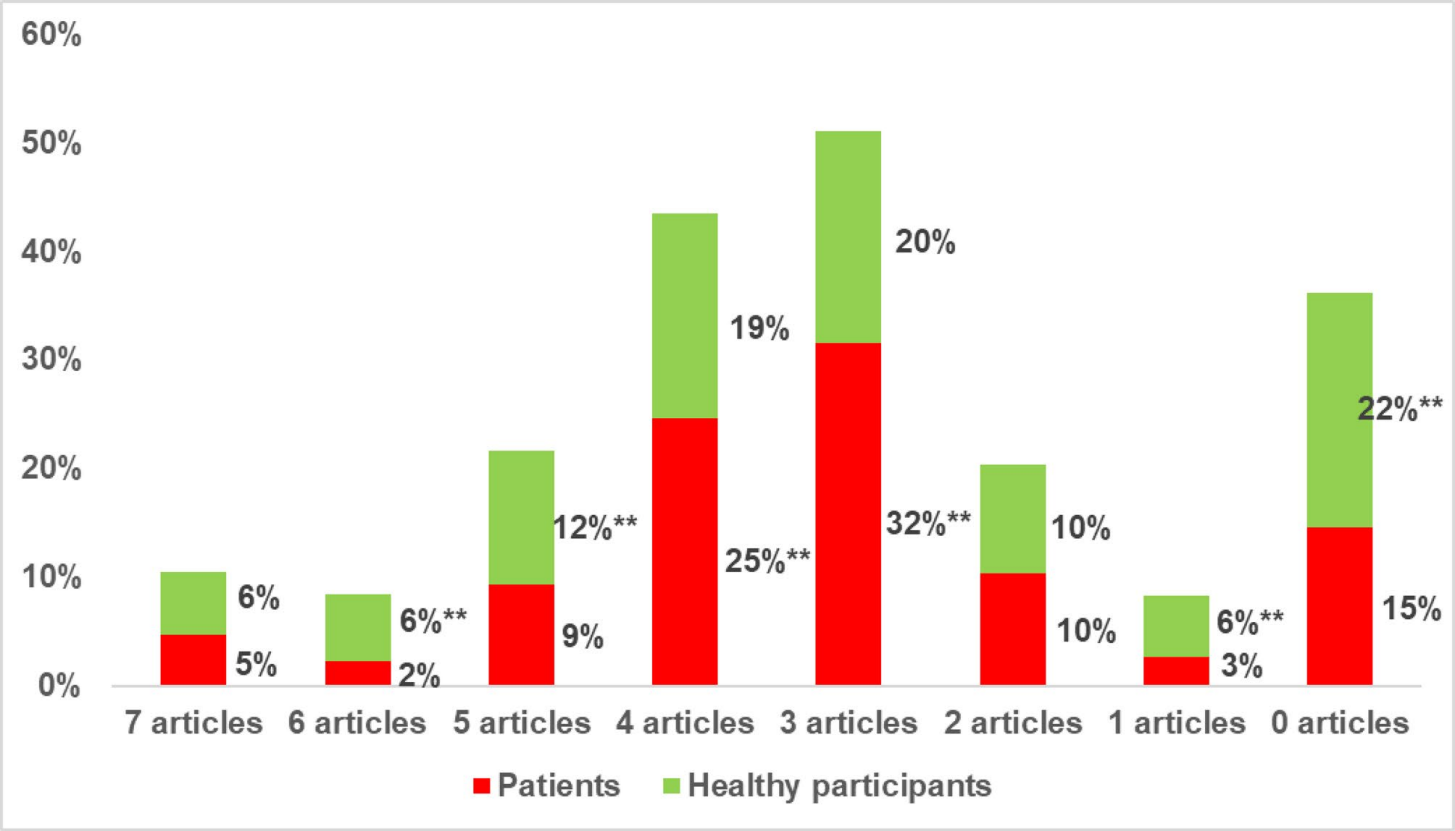
Awareness about Egyptian articles governing organ donation among the studied groups. Test of significant was z test between proportions, ** Highly significant: *p* < 0.01.

Awareness of Egyptian laws governing organ donation (Fig. 2) showed that 22% of healthy participants were unaware of any relevant articles, compared to 15% of patients. Additionally, 45% of patients were aware of fewer than four articles, in contrast to 35% of healthy participants.

Table 2 provides valuable insights into how patients with chronic illness and healthy participants view different consent options for posthumous organ donation. Written consent was the preferred method in both groups (75.2% of healthy participants vs. 70.1% of patients, *p* < 0.001). Patients were more likely to prefer oral consent (29.9% vs. 24.8%, *p* < 0.001). Patients were more likely to favor incentives for donation (59.6% vs. 54.7%, *p* < 0.001). Healthy participants preferred informed consent by the donor alone (12.2%) compared to patients (9.1%) (*p* < 0.001). Patients preferred surrogate consent (10.1%), oral consent (9.1%), and presumed consent (8.3%) at higher rates than healthy participants (5.8%, 5.0%, 4.5% respectively, *p* < 0.001).

**Table 2:** Comparison between patients with chronic illness and healthy participants regarding personal preferences for consenting posthumous organ donation.

Personal Preferences	Patients out of willing (*n* = 2739)	Healthy participants out of willing (*n* = 1815)	COR (95%CI)	*P* value by z test between proportions
Written consent	1920 (70.1)	1365 (75.2)	OR = 1	< 0.001**
Oral consent	819 (29.9)	450 (24.8)	0.8 (0.7–0.9)**	< 0.001**
**Personal Preferences of consent with or without incentives**				
With incentives	1632 (59.6)	993 (54.7)	OR = 1	< 0.001**
Without incentives	1107 (40.4)	822 (45.3)	1.2 (1.1–1.4)**	< 0.001**
**Personal Preference for Four Organ Donation Consenting Systems**				
Informed consent by donor only	249 (9.1)	221 (12.2)	OR = 1	< 0.001**
Informed consent by donor only or surrogate	277 (10.1)	105 (5.8)	0.4 (0.3–0.6)**	< 0.001**
Oral consent by donor only	249 (9.1)	91 (5.0)	0.4 (0.3–0.6)**	< 0.001**
Implementation of presumed consent	227 (8.3)	82 (4.5)	0.3 (0.3–0.5)**	< 0.001**

COR: Crude odds ratio, CI 95%: confidence interval, test of significance: X^2^ test, **p-value highly significant at < 0.01.

**Fig. 3:**
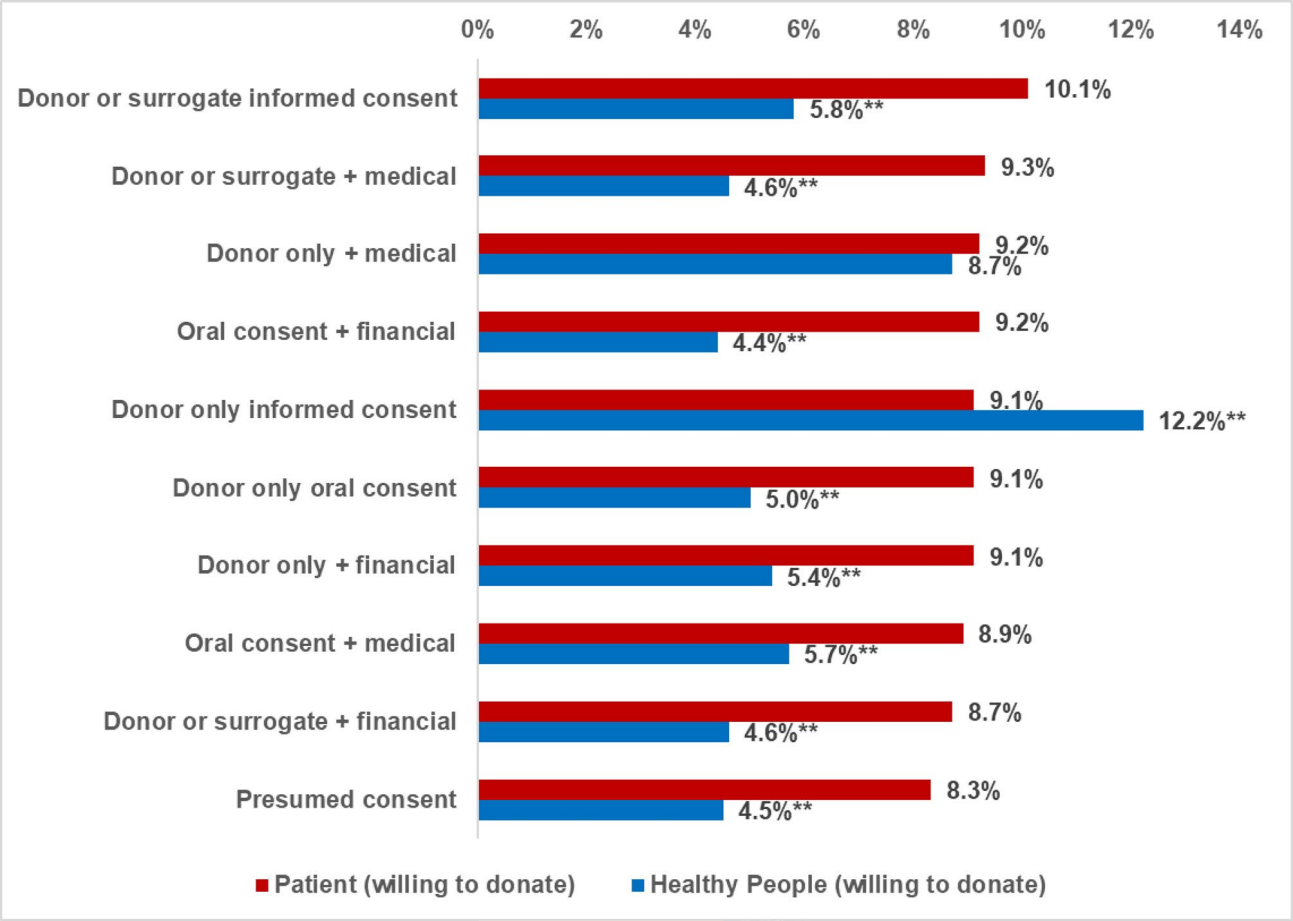
Personal preferences of the eleven consenting options among both groups. Test of significant was: z test between proportions, ** Highly significant: *p* < 0.01.

**Consenting options preference is shown in** Fig. 3. When participants of both groups were asked about the eleven consenting options of organ donation, patients significantly preferred almost all these consenting options compared to healthy participants except for informed consent by donor which was significantly preferred by both.

### Factors affecting the willingness to donate (Table 3)

The logistic regression analysis in Table 3 examines factors influencing willingness to donate organs posthumously among patients with chronic conditions and healthy participants. Although minor specific factors were found to be significantly affecting the patients’ willingness for posthumous donation, some factors were found to be significantly affecting willingness for posthumous donation among healthy participants.

### Key predictors for patients with chronic illness

Among patients with chronic illness, Age played a significant role, with individuals aged 45–65 years being significantly less likely to donate compared to those over 65 years (AOR = 0.29; 95%CI: 0.48–0.99, *p* = 0.04). Employment status influenced donation willingness, with students being significantly less likely to donate than other employment groups (AOR = 0.11; 95%CI: 0.02–0.65, *p* = 0.012). Job sector had a significant impact, as patients in professional, technical, or managerial roles were significantly more likely to donate than manual laborers (AOR = 9.37; 95%CI: 1.89–46.31, *p* = 0.006).

Socioeconomic class and awareness of organ donation laws did not significantly affect the willingness to donate among patients.

### Key predictors for healthy participants

- Age had a strong influence on willingness to donate, with participants aged 25–45 years (AOR = 2.49; 95%CI: 1.09–5.69, *p* = 0.030) and those younger than 25 years (AOR = 3.08; 95%CI: 1.23–7.75, *p* = 0.017) being significantly more likely to donate compared to those over 65 years.
- Employment status was a significant predictor, as retired individuals (AOR = 2.39; 95%CI: 1.42–4.02, *p* < 0.001), students (AOR = 2.20; 95%CI: 1.37–3.54, *p* < 0.001), and employed individuals (AOR = 5.99; 95%CI: 1.74–20.65, *p* < 0.001) were all more likely to donate compared to the unemployed group. Job sector revealed an inverse trend compared to patients, as professionals among healthy participants were significantly less likely to donate compared to manual workers (AOR = 0.51; 95%CI: 0.34–0.75, *p* < 0.001). Socioeconomic class had a notable effect, as individuals in higher socioeconomic classes (C, B, A) were significantly less likely to donate than those in the lowest SES category (D) (*p* < 0.001 for all).
- Among healthy participants, awareness of organ donation laws was strongly associated with willingness to donate. Participants aware of 1–3, 4–6, and all 7 key law articles were significantly more willing to donate, with AORs of 3.74; 95%CI: 2.81–4.99, 4.41; 95%CI: 3.17–06.14, and 5.22; 95%CI: 2.90–9.39, respectively (all *p* < 0.001). The acceptance of 7 law articles significantly increased the willingness to donate among them.
- Sex and education level are not significant predictors of the willingness to donate among either patients or healthy participants (*p* > 0.05 for both groups).

**Table 3:** Logistic regression model for factors affecting willing for posthumous organ donation among patients and healthy participants.

Variables	Patients			Healthy participants		
	β ^a^	AOR (95%CI)	*P* value	β ^a^	AOR (95%CI)	*P* value
**Sex (** *Male* **)**	0.19	1.20 (0.48–3.02)	0.69	−0.23	0.79 (0.62–1.04)	0.09
**Age categories** : Age > 65 (reference)						
Age 45-<65 years	−1.23	0.29 (0.09–0.99)	0.04*	0.11	1.12 (0.49–2.57)	0.79
Age 25-<45 years	−0.75	1.06 (0.12–1.85)	0.28	0.91	2.49 (1.09–5.69)	0.03*
< 25 years	−0.76	0.47 (0.04–5.88)	0.56	1.13	3.08 (1.23–7.75)	0.02*
**Education**: No formal education (reference)						
Primary/Preparatory	0.17	1.18 (0.56–2.49)	0.66	−0.29	0.75 (0.31–1.81)	0.52
Secondary	1.22	3.39 (0.99–11.55)	0.05	−0.31	0.73 (0.32–1.67)	0.46
University/Postgraduate	0.61	1.83 (0.56–5.95)	0.31	−0.28	0.76 (0.33–1.75)	0.52
**Employment**: Unemployed (reference)						
Retired	−0.49	0.61 (0.06–6.02)	0.68	0.87	2.39 (1.42–4.02)	< 0.001**
Student	−2.23	0.11 (0.02–0.65)	0.02*	0.79	2.20 (1.37–3.54)	< 0.001**
Employed	−0.89	0.41 (0.11–1.57)	0.19	1.79	5.99 (1.74–20.65)	< 0.001**
**Job**: Manual labour/construction (reference)						
Public sector	1.46	4.31 (0.72–25.91)	0.11	0.03	1.03 (0.61–1.74)	0.92
Professional technical managerial	2.24	9.37 (1.89–46.31)	< 0.001**	−0.67	0.51 (0.34–1.75)	< 0.001**
**Socioeconomic class**: Class D (reference)						
Class C	−0.29	0.75 (0.40–1.38)	0.35	−1.01	0.36 (0.27–0.49)	< 0.001**
Class B	−0.60	0.55 (0.05–5.64)	0.61	−1.24	0.29 (0.18–0.45)	< 0.001**
Class A	−0.49	0.61 (0.05–7.50)	0.70	−1.48	0.23 (0.15–0.35)	< 0.001**
**Awareness about law articles**: Awareness about 0 law articles (reference)						
Awareness about 1–3 law articles	−0.44	0.65 (0.28–1.50)	0.31	1.43	4.17 (3.14–5.54)	< 0.001**
Awareness about 4–6 law articles	−0.10	0.90 (0.35–2.36)	0.83	1.36	3.91 (2.85–5.37)	< 0.001**
Awareness about 7 law articles	−0.57	0.57 (0.13–2.45)	0.45	1.52	4.57 (2.55–8.19)	< 0.001**
**Constant**			0.000			0.906

Variable(s) entered in the model: Sex, age, education, job, socioeconomic class, awareness about law articles.

β: Regression coefficient, AOR: Adjusted Odds Ratio, CI = Confidence Interval, ^a^Analyzed using enter multivariate logistic regression, *Statistically significant at *p* < 0.05, **Statistically significant at *p* < 0.01.

## Discussion

Public trust in organ donation in Egypt is shaped by concerns over illegal organ trafficking and diverse Islamic interpretations of posthumous donation. While some scholars support donation during life, others oppose it after death due to theological debates about the soul’s departure. These challenges highlight the need for transparent policies and culturally sensitive awareness efforts to promote legitimate organ donation practices^12,29^.

### Willingness to donate: patients vs. Healthy individuals

“Willingness to donate posthumously was notably higher among patients than healthy individuals, and this willingness among patients appeared largely independent of socio-demographic factors; except for a reduced likelihood in those aged 45–65 years. In contrast, healthy individuals’ willingness was influenced by age, employment status, and legal awareness. Younger and employed participants were significantly more willing to donate, while higher socioeconomic status was associated with reduced willingness. Patients were also more open to surrogate consent, presumed consent, and incentivized models. Finally, legal awareness significantly increased donation willingness among healthy participants (*p* < 0.001) but had no effect on patients.

Our results suggest that chronic illness fosters an intrinsically stable willingness to donate, while healthy individuals’ attitudes are more shaped by external socio-demographic factors and legal awareness^30^. These findings reinforce the importance of structured legal education campaigns^2,31,32^.

The integration of TPB and HBM provides a useful lens for interpreting these group differences. TPB emphasizes the influence of subjective norms, perceived behavioral control, and attitudes toward behaviour; elements clearly reflected in healthy participants’ preferences for written consent, legal awareness, and autonomy^18,33^. HBM, on the other hand, highlights the role of perceived severity, susceptibility, and cues to action. All of which are especially relevant for patients whose illness heightens perceived need and benefits associated with donation^19^. The data from Table 3 supports this theoretical distinction: among healthy individuals, willingness was significantly influenced by employment (AOR = 5.99), legal awareness (AOR up to 4.57), and SES (AOR for Class A = 0.23), indicating the role of external control and social expectations (TPB). These associations highlight the predictive value of legal and social cues for individuals without chronic illness. The stronger effect of legal awareness among healthy participants highlights the importance of cues to action, supporting prior research showing that awareness of organ donation legislation strongly predicts willingness to donate in Egypt^9^.

Conversely, among patients, donation willingness was minimally influenced by external socio-demographic variables, reinforcing the HBM premise that perceived health vulnerability and intrinsic benefit perception override external factors.

Additional support for this divergence is seen in preference patterns from Table 2. Healthy participants showed a higher preference for written consent and donor-only models, reflecting TPB’s constructs of control and subjective norms. Meanwhile, patients were more open to surrogate and presumed consent, suggesting a utilitarian and experience-driven view consistent with HBM.

### The role of chronic illness in donation willingness (Patients vs. Healthy Individuals)

A notable finding was the significantly higher acceptance of posthumous donation among patients compared to healthy participants (91.0% vs. 60.0%, *p* < 0.01). This discrepancy may reflect patients’ personal encounters with chronic disease, which could enhance their empathy toward others awaiting transplants and deepen their appreciation of donation’s life-saving value. Additionally, the higher awareness of organ donation laws among patients (85% aware of at least one article) compared to healthy participants (78% aware) suggests that personal health challenges may drive individuals to seek more information about organ donation. This is consistent with international patterns, where individuals with personal or familial illness tend to report higher donation willingness^29^.

Our findings further demonstrate that donation willingness among patients is largely stable and internally driven, remaining unaffected by age, employment, or socioeconomic status (*p* > 0.05). This supports the notion that personal health experiences shape donation attitudes more strongly than external demographic variables. A study focusing on individuals with stage 5 chronic kidney disease (CKD) found that these patients generally held positive attitudes toward organ donation, and their willingness to donate was not significantly affected by socio-demographic variables. Similarly, our findings echo prior reports indicating that individuals with end-stage organ failure often maintain stable, favorable attitudes toward donation across diverse demographic groups^9,29,34^.

### Influence of Socio-Demographic factors on willingness (Age, job Status, SES, legal Awareness)

The logistic regression analysis in this study identified several factors significantly affecting the willingness to donate among healthy participants, including age, education, occupation, and awareness of organ donation laws. In contrast, patients’ willingness to donate appeared largely unaffected by socio-demographic characteristics, suggesting that personal health experiences may play a more dominant role than external influences.

Among patients, older adults (> 65 years) were more likely to express willingness, whereas those aged 45–65 years were less inclined to donate. This pattern may reflect age-related variations in health concerns, risk perception, or trust in medical institutions. Although a study among university students reported better knowledge and positive attitudes in younger individuals^35^, age was not a statistically significant predictor among patients, suggesting a more uniform outlook across age groups^36–38^.

In healthy participants, younger age groups (< 25 and 25–45 years) showed significantly greater willingness to donate, consistent with international trends showing higher donation willingness in younger populations^39^.

Education level was not a strong predictor for either group, except for a borderline significant effect in patients with secondary education. This implies that *general* awareness and targeted messaging may be more effective than formal education alone. Although one Egyptian study found higher knowledge among medical students compared to non-medical peers^35^, international evidence from countries like Spain shows public campaigns to be more impactful than educational attainment alone^39^. Students among patients were significantly less willing to donate, possibly reflecting limited engagement with chronic illness care systems. Among healthy individuals, being employed or retired strongly predicted donation willingness, potentially due to greater financial and social stability. Similar patterns are observed globally; for instance, employed individuals in the U.S. are more likely to be registered donors^39^, pointing to broader socioeconomic determinants of donation attitudes^37,38^.

Interestingly, professional workers among patients were more likely to donate, while professionals among healthy participants were less likely. This may reflect greater empathy or healthcare exposure among chronically ill professionals, while healthy professionals could harbor more skepticism or concerns about medical risks. This dichotomy is observed in various countries, where healthcare professionals exhibit higher donation rates compared to other professions^39^.

For healthy participants, individuals in higher socioeconomic classes were significantly less likely to be willing to donate compared to the lowest class (D). This may suggest that wealthier individuals feel less urgency to donate or harbor greater distrust toward the healthcare system. In contrast, individuals from lower SES backgrounds may view organ donation as a meaningful social contribution or equalizer, a dynamic also reported in Brazil and other middle-income countries^40^.

Among healthy participants, awareness of organ donation laws was strongly associated with willingness to donate. Legal knowledge may reassure individuals about ethical safeguards and procedural transparency, thereby increasing trust in the system.

Sex was not a significant predictor in either group. This is in line with previous Egyptian findings^35^ and broader global literature indicating that gender has limited impact on organ donation attitudes^39,41^.

This consistency across diverse populations highlights the importance of societal and cultural factors in shaping attitudes toward organ donation, regardless of individual characteristics. Such findings emphasize the need for targeted public education campaigns that address these shared influences to enhance donation rates globally^42^. In Egypt, this may indicate that cultural and religious values exert a stronger influence than education alone in shaping donation attitudes.

#### Consent preferences and implications for policy (Written Consent, surrogate Involvement, Incentives)

The results suggest that patients with chronic illness may have a more flexible and pragmatic approach to consent options, potentially due to their own experiences with illness and medical systems. In contrast, healthy participants favored formal, written consent and were less accepting of surrogate or presumed consent models. Incentivized consent appeals to both groups, with a slightly stronger preference among patients.

The observed preference for written consent (75.2% among healthy participants and 70.1% among patients) mirrors global trends. Countries such as Spain and the United States, which have implemented structured consent protocols, tend to achieve higher donation rates. This preference among healthy individuals may reflect a desire for control and clarity in decision-making, reinforcing the perceived importance of autonomy in this group. Spain’s opt-out system exemplifies how structured legislative models can enhance donation rates^43,44^.

Incentivized consent was favored by both groups, with patients showing a significantly stronger preference (59.6% vs. 54.7%, *p* = 0.001). This may stem from the financial burdens associated with chronic illness, as incentives could offer practical relief and enhance motivation. These findings support global literature suggesting that financial or priority-based incentives can increase donation rates, although ethical concerns persist regarding the potential erosion of altruism^32^.

The preference for informed consent among healthy participants (12.2%) compared to patients (9.1%) supports the interpretation that healthy individuals prioritize autonomy, while patients anticipating possible incapacitation lean toward surrogate involvement. This distinction underscores how health status influences the perceived value of individual agency versus delegated decision-making^36^.

Surrogate consent was notably more acceptable to patients (10.1%) than healthy participants (5.8%), consistent with research showing that family involvement is critical in health crises. Patients may view surrogate consent as a practical necessity, given the unpredictability of medical emergencies. Studies from countries with opt-out systems demonstrate significant increases in donation rates over time, reinforcing the importance of adaptable models^45^.

Oral consent by the donor only is more acceptable to patients than healthy participants. This greater acceptance may reflect a preference for simplified consent processes, shaped by patients’ routine engagement with healthcare systems. For patients, consent processes that are less formal and more straightforward might seem more manageable, especially given the challenges they already face with their health^46^.

Finally, the preference for presumed consent among patients (8.3%) compared to healthy participants (4.5%) suggests a more utilitarian approach to organ donation, likely influenced by their medical experiences. This aligns with findings from Rithalia et al. (2009), which reported that presumed consent systems can increase donation rates by 25–30% in various countries. Additionally, a systematic review indicated that countries with presumed consent legislation see an increase of approximately 2.7 to 6.14 more donors per million population; These data emphasize the potential of presumed consent to improve organ availability, particularly when aligned with patient perspectives. This contrasts with healthy individuals, highlighting the policy potential of default-based consent models^36–38^.

Together, these findings highlight the need for nuanced, context-sensitive consent frameworks that account for health status, autonomy concerns, and economic realities. Policy efforts should consider the acceptability of different consent models among key subgroups and balance ethical imperatives with practical strategies to increase organ availability.

#### Awareness and actionable consent: international and local comparisons

In this study awareness of donation laws among patients had no significant effect on their willingness to donate suggesting that their motivation to donate was intrinsic rather than influenced by legal knowledge. This supports the notion that chronic illness fosters an internalized commitment to donation. The study findings align with and expand upon international research on organ donation. The 91% willingness rate among Egyptian patients is significantly higher than the Middle Eastern average of 49.8% reported by Mekkodathil et al. (2020)^47^. This suggests that chronic illness plays a significant role in shaping positive attitudes toward organ donation in Egypt, potentially surpassing regional norms.

In contrast, willingness rates in the U.S. are higher (77%)^48^, likely due to presumed consent legislation and robust public awareness efforts. Similarly, Saudi Arabia reports a 67% willingness rate^49^, suggesting that targeted public education efforts may contribute to increased acceptance. The elevated acceptance rate among patients in our study may reflect growing normalization of donation as a necessary and altruistic act, despite persistent cultural or systemic challenges.

Among healthy participants, however, greater awareness of organ donation laws was strongly associated with increased willingness to donate. This highlights the relevance of the Theory of Planned Behavior, particularly the roles of perceived behavioral control and cues to action. The strong association between legal awareness and donation willingness (*p* < 0.001) supports findings from other regions, which emphasize that transparency and clarity in legal frameworks foster public trust^39,50^.

On the other hand, a study published in BMC Medical Ethics assessed Egyptians’ preferences regarding consenting options for posthumous organ donation, as well as their awareness and acceptance levels. The study found that while a majority of respondents (95.1%) had heard of organ donation, only a minority had taken steps to document their consent^2^. Similarly, a second study identified a parallel gap between awareness and action: despite high awareness, few individuals had made formal arrangements or registered as donors^51^.

Despite high awareness levels, various factors such as religious beliefs, mistrust in the medical system, and fear of body mutilation contribute to the reluctance toward organ donation^51^. Understanding these culturally rooted barriers is essential for developing nuanced, sensitive campaigns that not only raise awareness but also facilitate action.

## Strengths

This study is robust, with notable strengths including a large and representative sample size, a well-structured sampling strategy, and comprehensive data collection methods. The use of stratified random sampling and a mix of structured interviews and self-administered questionnaires ensures diverse demographic representation and accommodates participants’ literacy levels. Additionally, the clear explanation of data analysis methods, including logistic regression and χ²-tests, adds rigor and validity to the study’s findings.

Beyond methodological strengths, this study makes several novel contributions to the Egyptian literature on organ donation. It is the first to compare posthumous donation attitudes and predictors between patients with chronic illness and healthy individuals in a large, representative sample. Prior Egyptian studies have focused primarily on narrow subgroups (e.g., students or healthcare workers), whereas this research captures the broader societal spectrum. Furthermore, the application of an integrated TPB–HBM theoretical framework provides a comprehensive understanding of both intrinsic (e.g., health status) and extrinsic (e.g., legal awareness, perceived norms) drivers of donation attitudes and consent preferences. This dual-model approach offers a deeper behavioral interpretation than previously available in national research. By contextualizing donation behavior within Egypt’s health system and sociocultural norms, the study provides a critical knowledge base for evidence-informed policy, public education, and legislative strategies.

## Limitations

This study has several limitations that should be acknowledged. First, the study was conducted in only two governorates, which may limit the generalizability of the findings to the broader Egyptian population. Second, the cross-sectional nature of the study prevents the establishment of causality between socio-demographic variables and willingness to donate organs. Additionally, self-reported data may introduce social desirability bias, where participants may provide responses that align with perceived societal expectations rather than their true beliefs. Moreover, while the study employed the Health Belief Model (HBM) to interpret internal motivations for organ donation, it primarily focused on the core constructs such as perceived susceptibility, severity, benefits, and barriers. Two additional HBM constructs—‘cues to action’ (e.g., media campaigns, physician recommendations) and ‘self-efficacy’ (individuals’ confidence in their ability to become donors)—were not assessed in this analysis. Their exclusion may limit the full explanatory potential of the model. Future intervention research could benefit from exploring these dimensions, particularly given their relevance to designing targeted, behaviorally informed donation campaigns.

### Conclusion and recommendations

This study sheds light on the complex interplay between health status, socio-demographic influences, and legal awareness in shaping posthumous organ donation attitudes in Egypt. Patients with chronic illness demonstrated consistently higher willingness to donate, while healthy individuals’ attitudes were more sensitive to external factors such as age, employment, socioeconomic class, and familiarity with the law.

When interpreted through the lens of behavioral theory, our findings offer important insights. The heightened willingness to donate among chronically ill patients aligns with key constructs of the Health Belief Model (HBM); particularly perceived susceptibility to illness and perceived benefits of helping others through donation. Their internal health experiences appear to act as cues that heighten altruistic behavior. In contrast, healthy individuals’ willingness was more strongly associated with external influences such as social norms, perceived control over donation processes, and legal knowledge; core constructs of the Theory of Planned Behavior (TPB). This dual-theoretical interpretation underscores the added value of using an integrated TPB–HBM framework to understand organ donation decision-making.

Based on this integrated understanding, we recommend the adoption of multifaceted interventions that address both internal motivations and external barriers. For instance, public education campaigns should not only raise awareness about the health and societal benefits of donation (HBM) but also target social approval and perceived ease of taking action (TPB)—especially among younger and socioeconomically advantaged groups.

Further research should explore additional constructs such as self-efficacy and cues to action—components of HBM that were not assessed in the current study but may offer additional explanatory value. In policy terms, any shift toward presumed consent or incentive-based systems must be informed by robust public engagement, ensuring cultural alignment and respect for personal autonomy. Lastly, empowering healthcare professionals with culturally sensitive training to discuss organ donation at critical moments, including end-of-life care, could enhance informed decision-making and build trust in ethical donation practices.

These insights suggest that increasing organ donation rates requires a multifaceted approach. Public education campaigns; especially those tailored to middle-aged and higher socioeconomic groups can help address misconceptions and foster trust. Prior successful community-based initiatives^52–55^ and social marketing strategies in Egypt^56–59^ offer effective models for such efforts.

## Data Availability

The datasets used and analyzed for the current study are available from the corresponding author upon reasonable request.
